# Synthesis of Hydrotalcites from Waste Steel Slag with [Bmim]OH Intercalated for the Transesterification of Glycerol Carbonate

**DOI:** 10.3390/molecules25194355

**Published:** 2020-09-23

**Authors:** Guanhao Liu, Jingyi Yang, Xinru Xu

**Affiliations:** International Joint Research Center of Green Energy Chemical Engineering, East China University of Science and Technology, Meilong Road 130, Shanghai 200237, China; jyyang@ecust.edu.cn (J.Y.); xrxu86@ecust.edu.cn (X.X.)

**Keywords:** steel slag, hydrotalcites, ionic liquid, transesterification

## Abstract

Ca-Mg-Al hydrotalcites were prepared by coprecipitation from Type S95 steel slag of Shanghai Baosteel Group as supports of ionic liquid in this paper. Five basic ionic liquids [Bmim][CH_3_COO], [Bmim][HCOO], [Bmim]OH, [Bmim]Br and ChOH were prepared and their catalytic performance on the synthesis of glycerol carbonate by transesterification between dimethyl carbonate and glycerol was investigated. The characterization results indicated that [Bmim]OH is the best ionic liquid (IL) for the transesterification reaction of glycerol carbonate. The hydrotalcites before and after intercalation by ionic liquid were characterized by XRD, FTIR, SEM, EDS and the IL were characterized by FT-IR, ^13^C-NMR and basicity determination via the Hammett method. The analysis results implied that the dispersion of [Bmim]OH in hydrotalcites reduced the alkali density appropriately and facilitated the generation of glycerol carbonate. The yield of glycerol carbonate and the conversion rate of glycerol reached 95.0% and 96.1%, respectively, when the molar ratio of dimethyl carbonate and glycerol was 3:1, the catalyst dosage was 3 wt%, the reaction temperature was 75 °C and the reaction time was 120 min. The layered structure of hydrotalcites increased the stability of ionic liquid intercalated in carriers, thus the glycerol conversion and the GC yield still remained 91.9% and 90.5% in the fifth reaction cycle.

## 1. Introduction

Steel slag is the solid waste composed of Ca, Si, Fe, Mg, Al, Mn, P, O and so on in the steel-making procedure [1]. The simple utilization of steel slag usually causes the loss of valuable metal resources due to the massive emissions [2]. The new door to the comprehensive utilization of steel slag will be opened up if the various metal resources can be used for the preparation of new multifunctional materials with high value [3,4].

Layered double hydroxides (LDHs), also known as hydrotalcite-like compounds, are anionic layered compounds with similar crystal structures to brucites [5]. The host layers and guest anions in LDHs are connected by noncovalent bonds. LDHs have broad application prospects in adsorption, catalysis, biomedicine and other fields due to their unique properties such as pore size tunability, interlayer anion exchangeability [6] and interlayer cation compatibility [7]. Specially, LDHs are often used as carriers in the catalysis field due to their layered structures [8].

Carbonate glycerol (GC) is widely used in food, medicine, plastics, military, new materials and new energy as a special bio-based chemical with high boiling point, low freezing point, low volatility, low flammability and strong polarity [9]. The transesterification of glycerol with dimethyl carbonate (DMC) over basic catalysts is considered to be an effective method for GC synthesis under mild conditions [10]. The only reaction byproduct methanol is easily separated from the product mixture. Meanwhile, toxic substances, relatively high reaction pressure and other harsh reaction conditions can be avoided in the process [11]. The preparation of a catalyst with high activity and stability is the necessary requirement for large-scale production of GC [12].

Ionic liquid has been of wide concern in the field of catalysis in recent years due to its mild reaction conditions, high-efficiency selectivity and simple operation process [13]. However, it is difficult to separate ionic liquid from the homogeneous system after reaction [14]. The immobilization of ionic liquid can not only retain remarkable catalytic performance, but also overcome the separation difficulties [15]. Meanwhile, new properties and characteristics of catalysts will be derived via the synergism between ionic liquid and carriers [16].

In this paper, alkaline ionic liquids with different structures were designed and synthesized for the transesterification of dimethyl carbonate and glycerol to produce glycerol carbonate. The effect of different ions on the catalytic ability was investigated. Ca-Mg-Al hydrotalcites were prepared from waste steel slag and used as carriers for the intercalation of ionic liquid. This process recycled the waste steel slag, reduced the synthesis cost of hydrotalcites and solved the separation problem of ionic liquid.

## 2. Results and Discussion

### 2.1. Characterization of Ionic Liquid

#### 2.1.1. ^13^C-NMR analysis of Ionic Liquid

**Figure 1:**
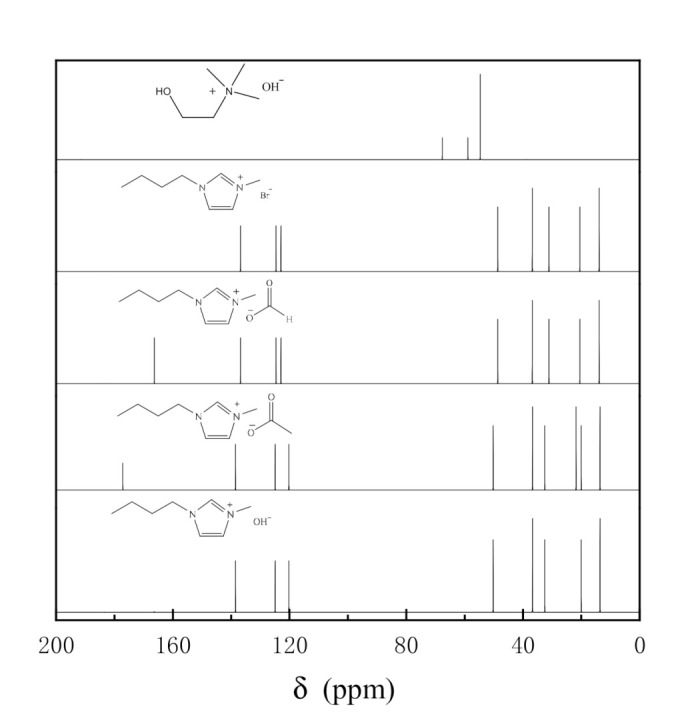
^13^C-NMR spectra of [Bmim]OH, [Bmim][CH_3_COO], [Bmim][HCOO], [Bmim]Br and ChOH.

Figure 1 showed the ^13^C-NMR spectra of [Bmim][CH_3_COO], [Bmim][HCOO], [Bmim]OH, [Bmim]Br and ChOH. The peaks at 67.68 ppm and 58.86 ppm were the resonance signals of methylene on quaternary ammonium cations, and the chemical shift of methyl on quaternary ammonium cations was 54.66 ppm in the ^13^C-NMR spectra of ChOH. In the other four spectra, the characteristic peaks at 138.80 ppm, 124.94 ppm and 120.24 ppm corresponded to the chemical shifts of carbon on the imidazole ring. The resonance signals of carbon on the methylene linked to the imidazole ring were 48.66 ppm, 31.10 ppm and 20.50 ppm. The peaks appeared where δ was 36.88 ppm, and 13.87 ppm can be attributed to the methyl carbon on imidazole ring branch. The carbon chemical shifts of acetate were 177.14 ppm and 21.83 ppm in the NMR spectrum of [Bmim][CH_3_COO]. The peak where δ was 166.35 ppm can be ascribed to the resonance signals of formate in the NMR spectrum of [Bmim][HCOO]. The four synthesized products had similar structure of 1-butyl-3-methyl imidazole cation while the anions were bromine, hydroxyl, acetate and formate, respectively, according to the above NMR analysis results.

#### 2.1.2. FT-IR Analysis of Ionic Liquid

**Figure 2:**
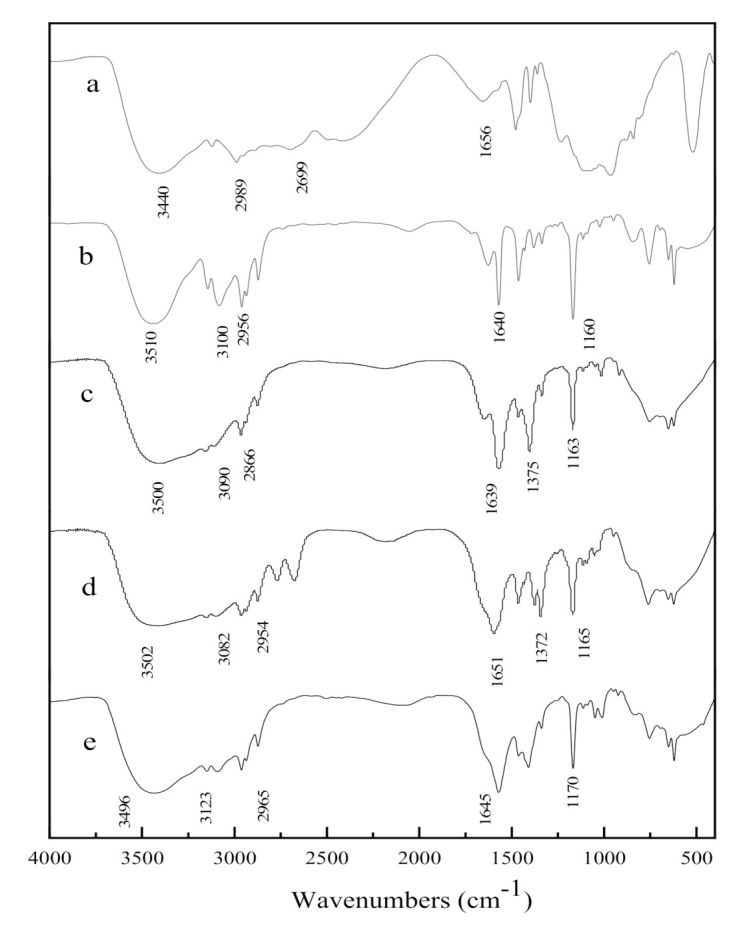
FT-IR spectra of ChOH (**a**), [Bmim]Br (**b**), [Bmim][HCOO] (**c**), [Bmim][CH_3_COO] (**d**) and [Bmim]OH (**e**).

The FT-IR spectra of different ionic liquids were displayed in Figure 2. The related characteristic absorption peaks of imidazole were shown in the spectra of [Bmim][CH_3_COO], [Bmim][HCOO], [Bmim]OH and [Bmim]Br. The stretching vibration peaks of unsaturated C-H on imidazole rings appeared at 3123 cm^−1^ and 3082 cm^−1^. The absorption peaks at 2965 cm^−1^, 2954 cm^−1^ and 2866 cm^−1^ were corresponded to the stretching vibration of saturated C-H bond on branch chain. The characteristic peaks at 1645 cm^−1^ and 1163 cm^−1^ were attributed to the stretching vibration of C-N and C=C on imidazole ring. Duan [17] reported similar results about the imidazolium cation. The absorption peak at about 3500 cm^−1^ was caused by the stretching vibration of hydroxyl. In the infrared spectra of [Bmim][CH_3_COO] and [Bmim][HCOO], the absorption peak at 1375 cm^−1^ was ascribed to the asymmetric stretching vibration of C-O in carboxylate. The absorption peak at 1656 cm^−1^ was corresponded to N-C stretching vibration of quaternary ammonium cations in the FT-IR spectra of ChOH. The stretching vibration peaks of methylene and methyl on the branched chain of quaternary ammonium cation appeared at 2989 cm^−1^ and 2699 cm^−1^. All the above indicated that the ionic liquid had been synthesized successfully.

#### 2.1.3. Results of Basicity of Ionic Liquids

The catalyst basicity was tested, and the influence on the reaction activity was studied in this section. The basic amount of [Bmim]Br, [Bmim][HCOO], [Bmim][CH_3_COO], ChOH and [Bmim]OH was 0.69 mmol/g, 0.87 mmol/g, 0.92 mmol/g, 1.09 mmol/g and 1.17 mmol/g, respectively, in Table 1. The glycerol conversion and the GC yield mounted up with the increase of basicity while the reaction selectivity decreased because glycerol carbonate was further decarboxylated to generate glycidol in the strong alkaline environment. The intercalation of ionic liquid in hydrotalcites reduced the alkali density appropriately, which not only helped to improve the selectivity, but also solved the separation problem of ionic liquid.

### 2.2. Characterization of IL-CaMgAl

#### 2.2.1. XRD Analysis

**Figure 3:**
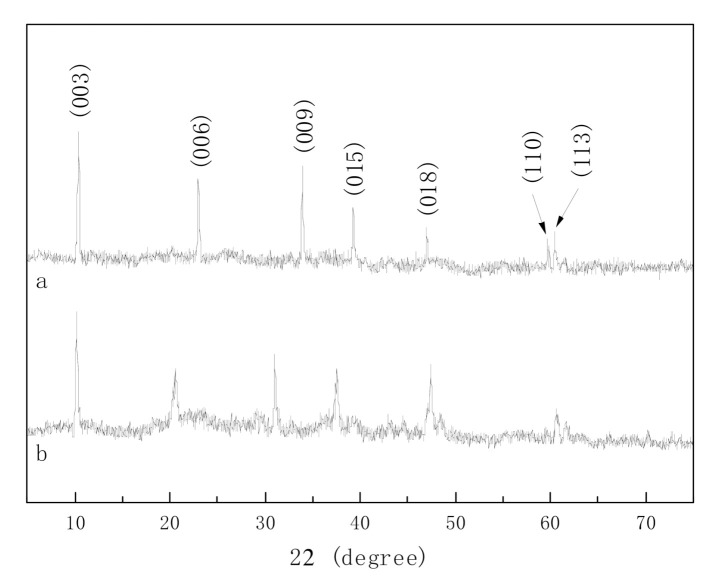
XRD patterns of pure hydrotalcites (**a**) and IL-CaMgAl (**b**).

Figure 3 was the XRD patterns of pure hydrotalcites and ionic liquid intercalated hydrotalcites. IL-CaMgAl still had the characteristic peaks of hydrotalcite lattice planes (003), (006), (009), (015), (018), (110) and (113) [6]. The position of each characteristic diffraction peak in IL-CaMgAl was slightly shifted but can be clearly distinguished compared with the XRD spectrum of pure hydrotalcites, which indicated that the intercalation of [Bmim]OH did not change the crystal structure of hydrotalcites.

#### 2.2.2. FT-IR Analysis

**Figure 4:**
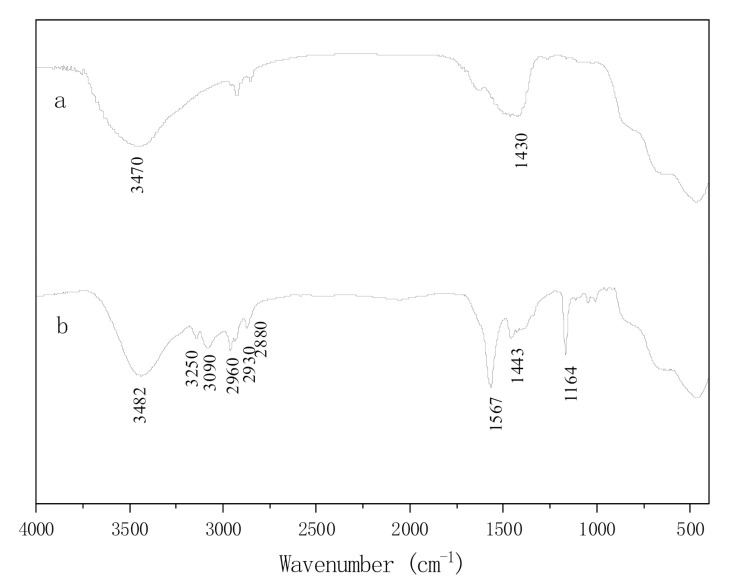
FT-IR spectra of pure hydrotalcites (**a**) and IL-CaMgAl (**b**).

FT-IR spectra of CaMgAl hydrotalcites before and after intercalation by ionic liquid were shown in Figure 4. The absorption peak of pure hydrotalcites at 3470 cm^−1^ was related to hydroxyl stretching vibration linked to metal cations. The absorption vibration peak of carbonyl in carbonates was observed at 1430 cm^−1^. The characteristic peaks of imidazole ionic liquid appeared, obviously, on the infrared spectrum of hydrotalcites after intercalation by [Bmim]OH. The absorption peaks at 3250 cm^−1^ and 3090 cm^−1^ corresponded to the unsaturated C-H on imidazole rings. The stretching vibration peaks at 2960 cm^−1^, 2930 cm^−1^ and 2880 cm^−1^ were attributed to the saturated carbon-hydrogen bond on the branch chain of imidazole rings. The absorption peaks at 1567 cm^−1^ and 1164 cm^−1^ were ascribed to C=N and C-C on imidazole rings, respectively. All indicated that [Bmim]OH had been intercalated into hydrotalcites successfully.

#### 2.2.3. Element Analysis

Table 2 was the main element analysis of pure hydrotalcites and IL-CaMgAl. The contents of oxygen, calcium, aluminum, carbon and magnesium in the pure hydrotalcites were 45.49 wt%, 28.74 wt%, 9.99 wt%, 6.11 wt% and 4.04 wt%, respectively, while the contents of oxygen, calcium, carbon, aluminum, nitrogen and magnesium in the IL-CaMgAl were 41.93 wt%, 27.53 wt%, 12.30 wt%, 7.98 wt%, 5.25 wt% and 3.66 wt%, respectively. The occurrence of nitrogen and the increase of carbon indicated that the ionic liquid had immobilized in the hydrotalcites since [Bmim]OH contained a large amount of carbon and nitrogen elements.

#### 2.2.4. SEM Analysis

**Figure 5:**
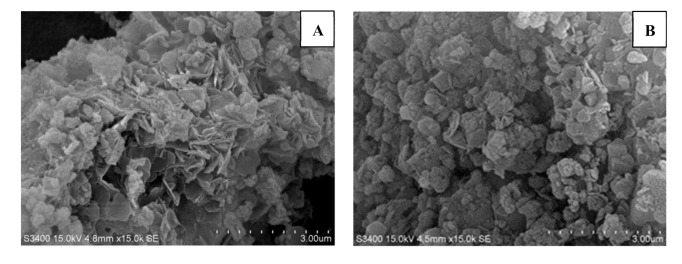
SEM images of pure hydrotalcites (**A**) and IL-CaMgAl (**B**).

The petaloid layered structures was the characteristic morphology of hydrotalcites [7]. The polygonal or circular lamellas can be seen clearly in the SEM images of pure hydrotalcites in Figure 5. The layered morphology was still maintained after intercalation of [Bmim]OH in the SEM images of IL-CaMgAl, but the lamellas were distorted to some extent.

### 2.3. Transesterification of Glycerol and Dimethyl Carbonate

**Figure 6:**
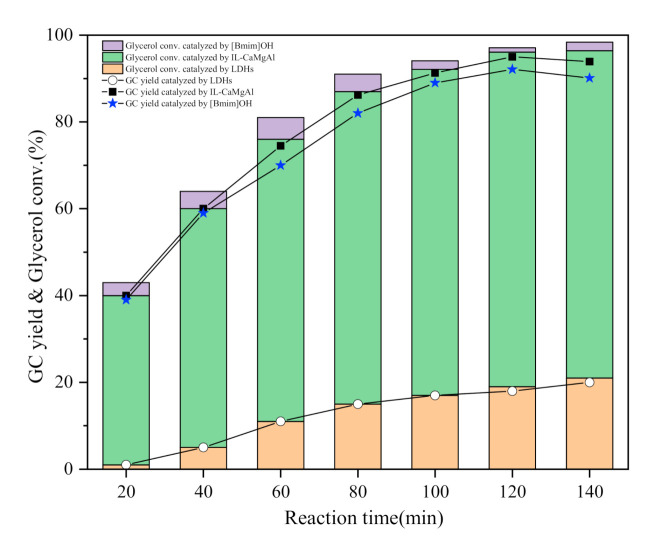
Transesterification of dimethyl carbonate and glycerol catalyzed by [Bmim]OH, layered double hydroxides (LDHs) and IL-CaMgAl.

The intercalation of [Bmim]OH with strong alkalinity changed the catalytic activity of hydrotalcites, obviously. The esterification of dimethyl carbonate and glycerol was carried out catalyzed by IL-CaMgAl and pure hydrotalcites respectively under the condition that the catalyst dosage was 3 wt%, the molar ratio of DMC and glycerol was 3:1, the reaction time was 120 min and the reaction temperature was 75 °C in order to investigate the influence of ionic liquid intercalation on esterification. The reaction results were presented in Figure 6.

The conversion rate of glycerol and the yield of glycerol carbonate catalyzed by IL-CaMgAl were much higher than that by pure hydrotalcites in the same reaction time. The glycerol conversion rate and the GC yield catalyzed by pure hydrotalcites were both at a low level all along. The conversion rate of glycerol catalyzed by ionic liquid intercalated hydrotalcites reached 60.1% without by-product when the reaction lasted for 40 min. The GC yield was 95.0% and the glycerol conversion was 96.1% when the reaction time was 120 min. However, the yield of GC decreased slightly although the conversion rate of glycerol increased when the reaction time continued to mount up since the further decarboxylation of glycerol carbonate generated glycidol in strong alkaline environment. The optimal reaction time was 120 min under the research conditions based on the above experimental results. The catalytic performance comparison of different catalysts was listed in Table 3.

### 2.4. Reusability Test of IL-CaMgAl

**Figure 7:**
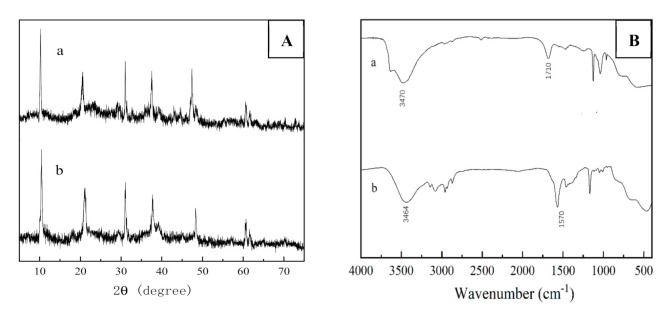
XRD patterns (**A**) and FT-IR spectra (**B**) of five times reused catalyst (**a**) and fresh catalyst (**b**).

Continuous industrial reaction required high stability of catalyst materials to resist rapid deactivation and leaching of active sites. The cycle experiment was carried out when the molar ratio of DMC and glycerol was 3:1, the catalyst dosage was 3 wt%, the reaction temperature was 75 °C and the reaction time was 120 min. IL-CaMgAl was washed with methanol after each reaction cycle. Figure 7 displayed the XRD patterns and FT-IR spectra of fresh catalyst and five times reused catalyst. The crystal morphology of catalyst after five cycles was almost identical to the fresh catalyst. However, the adsorption peak of [Bmim]OH became weak and the stretching vibration peak of ester group at 1710 cm^−1^ appeared in the FT-IR spectrum of five times reused catalysts, which indicated the loss of active components and the adhesion of organics.

**Figure 8:**
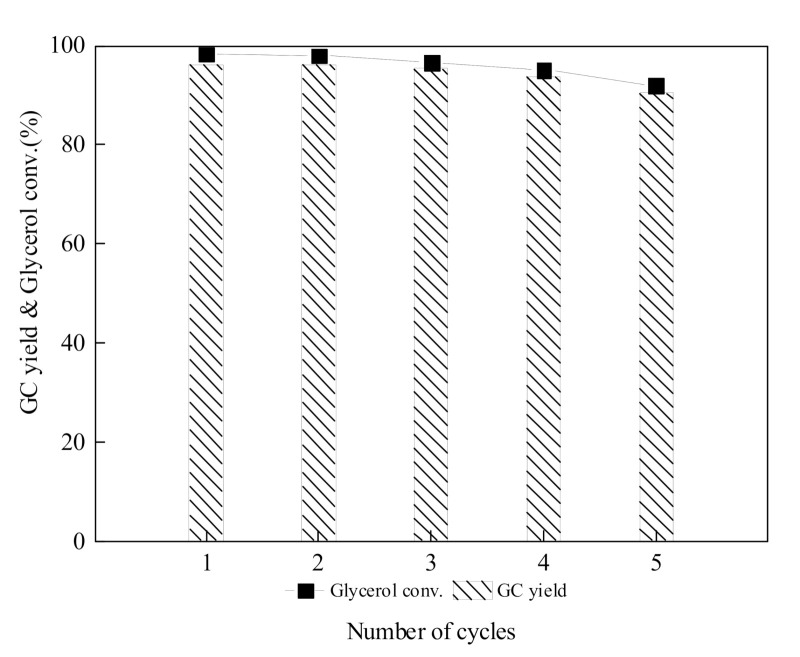
Reusability of IL-CaMgAl.

Figure 8 showed the change of catalytic activity of IL-CaMgAl in the five cycles. The catalyst remained 91.9% glycerol conversion rate and 90.5% GC yield in the fifth reaction cycle, which meant IL-CaMgAl had remarkable reusability in the continuous reaction. The hydrotalcites with layered structures can immobilized the ionic liquid and, thus, reduce the loss of active components in the catalyst.

## 3. Experimental

### 3.1. Preparation of Ionic Liquid

First, 1-methylimidazole (0.05 mol) was added into the four-neck flask with the condenser tube and heated slowly to 70 °C. Some *n*-butylbromide, in the constant pressure funnel, was added dropwise at 1:1.1 molar ratio of 1-methylimidazole to *n*-butylbromide. The reaction was carried out at 70 °C under the atmosphere of nitrogen. The yellowish intermediate [Bmim]Br was obtained after washing with ethyl acetate and drying in a vacuum oven at 70 °C for 24 h.

[Bmim]Br (0.03 mol) and potassium acetate were weighed at the molar ratio of 1:1 and dissolved in methanol. The reaction lasted for 8 h at 25 °C, 10 mL ether was added after reaction and the white precipitate was removed by filtration. The filtrate was evaporated for 1 h at 55 °C and dried in the vacuum oven at 70 °C for 24 h to obtain ionic liquid [Bmim][CH_3_COO]. [Bmim][HCOO] and [Bmim]OH can be prepared by changing the above-mentioned potassium acetate into potassium formate and sodium hydroxide, respectively.

The quaternary ammonium ionic liquid ChOH was synthesized by using choline chloride and sodium hydroxide as raw materials at the molar ratio of 1:1. Choline chloride and sodium hydroxide were weighed and dissolved in dichloromethane solution. The dichloromethane was removed by rotary evaporation after the reaction, and ChOH was obtained after washing with ethyl acetate, filtering and drying.

### 3.2. Preparation of Ca-Mg-Al Hydrotalcites Intercalated by [Bmim]OH

Hydrotalcites were synthesized by coprecipitation method in this study. The steel slag from Shanghai Baosteel Group was dissolved in 34 wt% nitric acid solution to prepare solution A. Appropriately, NaOH and Na_2_CO_3_, at the molar ratio of 1:16, was dissolved in deionized water to prepare solution B. Then, 50 mL deionized water and [Bmim]OH was add into a four-neck flask with stirring devices. Solution A and solution B were dropped by double-titration method. The system temperature was kept at 70 °C, and the pH value was kept between 10 and 11 during titration. The obtained white slurry was aged in a constant-temperature water bath at 70 °C for 24 h. The hydrotalcites intercalated by ionic liquid were synthesized after filtering, washing with deionized water and drying in the vacuum oven at 70 °C for 24 h. Pure hydrotalcites can be prepared without adding ionic liquid in the coprecipitation.

### 3.3. Characterization Methods

The crystallite sizes of solid catalyst were characterized by X-ray diffraction (XRD) on Rigaku D/max2550VB/PC (Rigaku International Corporation, Tokyo, Japan) with CuKα radiation (λ = 0.154 nm, 100 mA, 45 kV). The scanning range was 10–80°(2θ) with a rate of 0.02°/s. Surface elements were measured by Edax Falcon energy dispersive spectrometer (EDS) (EDAX Co., Ltd., Mahwah, NJ, USA). The morphologies of hydrotalcites was observed by scanning electron microscope (SEM, Hitachi S-3400N, Hitachi Co., Ltd., Tokyo, Japan). Fourier transform infrared (FT-IR) absorption spectra of the samples were determined by Nicolet Magna-IR 550 via KBr pellet (Thermo Fisher Scientific, Waltham, MA, USA). Avance 500 nuclear magnetic resonance spectrometer of Bruker company in Germany was used to determine the ^13^C-NMR spectrum of samples. Hammett indicators including 4-nitroaniline (H_ = 18.4), 2.4-dinitroaniline (H_ = 15.0), phenolphthalein (H_ = 9.8) and 4-chloroaniline (H_ = 26.5) were used to measure the basic strength of ionic liquid by various color. The ionic liquid was diluted with deionized water and titrated with hydrochloric acid standard solution using phenolphthalein as indicator. The alkali amount can be calculated by the amount of hydrochloric acid consumed.

### 3.4. Transesterification Procedure

Glycerol and dimethyl carbonate according to the molar ratio of 1:3 was added into the flask with the stirring device and reflux device. The mixture was heated to 75 °C under the atmosphere of nitrogen. Then, 3 wt% IL-CaMgAl was added to catalyze the transesterification between glycerol and dimethyl carbonate. The product was separated from solid catalyst after 120 min by centrifugation and analyzed by gas chromatography. The gas chromatograph (Jinghe GC-7860, Jing He Analysis Instrument Co., Ltd., Shanghai, China) was equipped with a flame ionization detector (FID) (Agilent Technologies Inc., Santa Clara, CA, USA) and a capillary column (HP-PONA, 50 m by 0.200 mm by 0.50 μm). The injection temperature and detector temperature were 325 °C and 280 °C, respectively.

## 4. Conclusions

The partial metal elements such as Ca, Mg and Al in waste steel slag can be utilized effectively by preparing the environment-friendly catalyst in this paper. The intercalation of ionic liquid [Bmim]OH adjusted the basicity of hydrotalcites and improved the GC yield in the transesterification of dimethyl carbonate and glycerol. The highest yield of glycerol carbonate can reach 95.0% under the condition that the molar ratio of DMC and glycerol was 3:1, the catalyst dosage was 3 wt%, the reaction time was 120 min and the reaction temperature was 75 °C. Meanwhile, the supported solid catalyst also solved the separation difficulty and loss problem of ionic liquid. The conversion rate of glycerol and the yield of GC still remained 91.9% and 90.5% after five cycles catalyzed by IL-CaMgAl. The significant reusability and high catalytic activity of IL-CaMgAl made it meet the requirements of continuous production and have extensive application perspective in industry. However, all experiments in this paper were carried out in a lab scale, thus the pilot-scale study was necessary for the further research. 

## Figures and Tables

**Table 1:** Effect of ionic liquid basicity on glycerol conversation and carbonate glycerol (GC) yield.

Catalyst	Glycerol Conv. (%)	GC Yield (%)	Basic Amount (mmol/g)	Basic Strength (H_)
[Bmim]HCOO	81.6	81.1	0.87	H_ < 9.8
[Bmim][CH_3_COO]	90.5	90.3	0.92	9.8 < H_ < 15.0
[Bmim]OH	97.5	92.1	1.17	18.4 < H_ < 26.5
[Bmim]Br	75.2	75.0	0.69	H_ < 9.8
ChOH	96.5	91.4	1.09	18.4 < H_ < 26.5

**Table 2:** Surface element content of CaMgAl and IL-CaMgAl.

Element (wt.%)	C	N	O	Mg	Al	Ca	Others
CaMgAl	6.11	NA *	45.49	4.04	9.99	28.74	5.63
IL-CaMgAl	12.30	5.25	41.93	3.66	7.98	27.53	1.35

* NA means the element content can not be detected by the instrument.

**Table 3:** Comparison of IL-CaMgAl with reported catalysts in transesterification of dimethyl carbonate (DMC) and glycerol.

Catalyst	Catalyst Dosage (wt.%)	Reaction Temperature (℃)	Molar Ratio of DMC to Glycerol	Reaction Time (min)	GC Yield (%)	Ref.
CaO	3	75	2:1	30	90.2	[18]
Na_2_SiO_3_-200	5	75	4:1	150	95.5	[19]
Calcined Dolomite	6	75	3:1	90	94.0	[20]
NaY zeolite	10	70	3:1	240	80.0	[21]
IL-CaMgAl	3	75	3:1	90	96.2	This work
